# Comparison of radar data versus rainfall data

**DOI:** 10.1016/j.mex.2015.10.007

**Published:** 2015-10-20

**Authors:** B. Espinosa, T.V. Hromadka, R. Perez

**Affiliations:** a Hromadka & Associates, 29809 Santa Margarita Parkway Suite 102, RSM, CA 92688, United States; b Department of Mathematical Sciences, United States Military Academy, West Point, NY 10996, United States

**Keywords:** Rainfall data accuracy check, Doppler radar, Ground-truth, Topographic interference, Radar, Calibration

## Abstract

Doppler radar data are increasingly used in rainfall-runoff synthesis studies, perhaps due to radar data availability, among other factors. However, the veracity of the radar data are often a topic of concern. In this paper, three Doppler radar outcomes developed by the United States National Weather Service at three radar sites are examined and compared to actual rain gage data for two separate severe storm events in order to assess accuracy in the published radar estimates of rainfall. Because the subject storms were very intense rainfall events lasting approximately one hour in duration, direct comparisons between the three radar gages themselves can be made, as well as a comparison to rain gage data at a rain gage location subjected to the same storm cells. It is shown that topographic interference with the radar outcomes can be a significant factor leading to differences between radar and rain gage readings, and that care is needed in calibrating radar outcomes using available rain gage data in order to interpolate rainfall estimates between rain gages using the spatial variation observed in the radar readings. The paper establishes and describes

• the need for “ground-truthing” of radar data, and
• possible errors due to topographic interference.

## Graphical abstract

**Figure:**
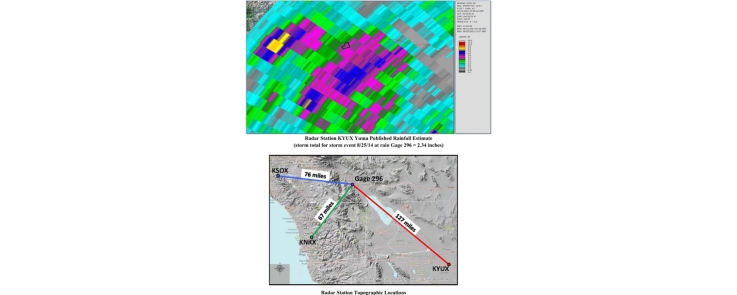

## Method details

Doppler radar information is increasingly being used to estimate rainfall quantities. However, while the graphical outcomes are attractive, the data values may be questionable. Care is needed to “ground truth” the radar data by adjusting it to conform to available rain gage data where possible. In this paper, three Doppler radar site outcomes for two severe 1-h duration rainstorms that occurred in Southern California, USA, are examined as to consistency in their rainfall estimates. Considerable variation is seen in the radar estimates for the same storm and location. Ground interference of the radar is suggested as a possible cause in this case, but the variation between rainfall data and radar estimates of rainfall is shown to vary considerably even where there is little to no topographic interference.

## Study location

**Fig. 1:**
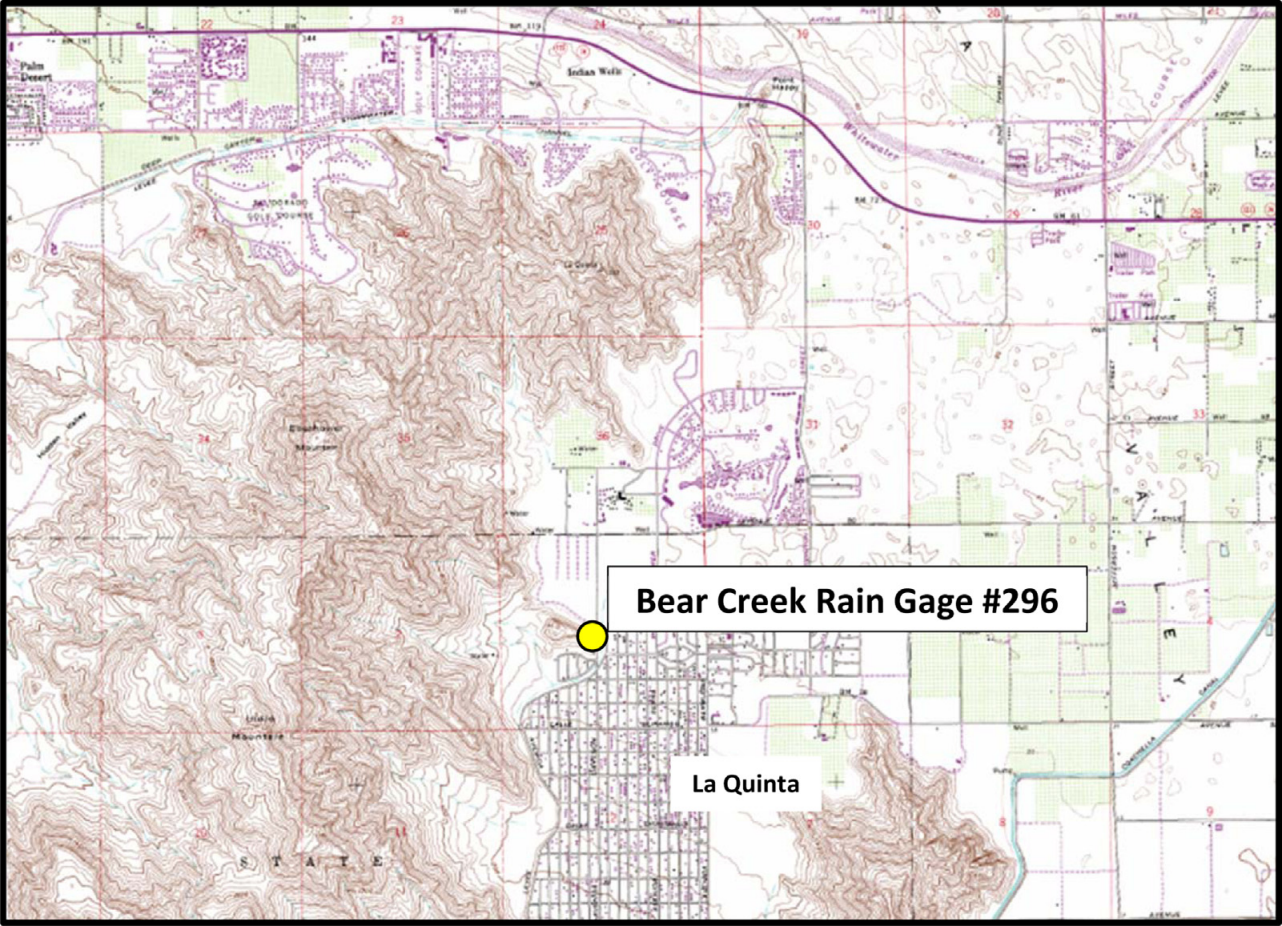
Subject rain gage location.

The study site is located in the City of La Quinta, CA, a desert environment municipality at the foot of the Santa Rosa Mountains on the floor of the Coachella Valley (see Fig. 1). Average monthly temperature highs range from 71° F in the winter to 107° F in the summer, and average annual rainfall is less than 5 in. [1]. The late summer rainfalls in the area usually come in the form of fast moving thunderstorms. The two storm events presented herein occurred on August 25, 2013 and September 8, 2014.

## Rain gage and Doppler radar sites

The subject rain gage for this study, Bear Creek Rain Gage #296 (see Fig. 1), is an ALERT gage operated by the Coachella Valley Water District (CVWD) [2]. An ALERT gage produces a continuous rainfall record by recoding the time at which a pre-selected amount of rainfall is collected in the gage. For example, if the gage records every time 0.04 in. of rainfall is collected, a heavy storm event may produce multiple time stamps during a five minute period, whereas a less intense storm event may only produce a single time stamp in five minutes (if any). The rain data for this gage for the two storms at issue is available as 5-min incremental rainfall accumulations [3].

**Fig. 2:**
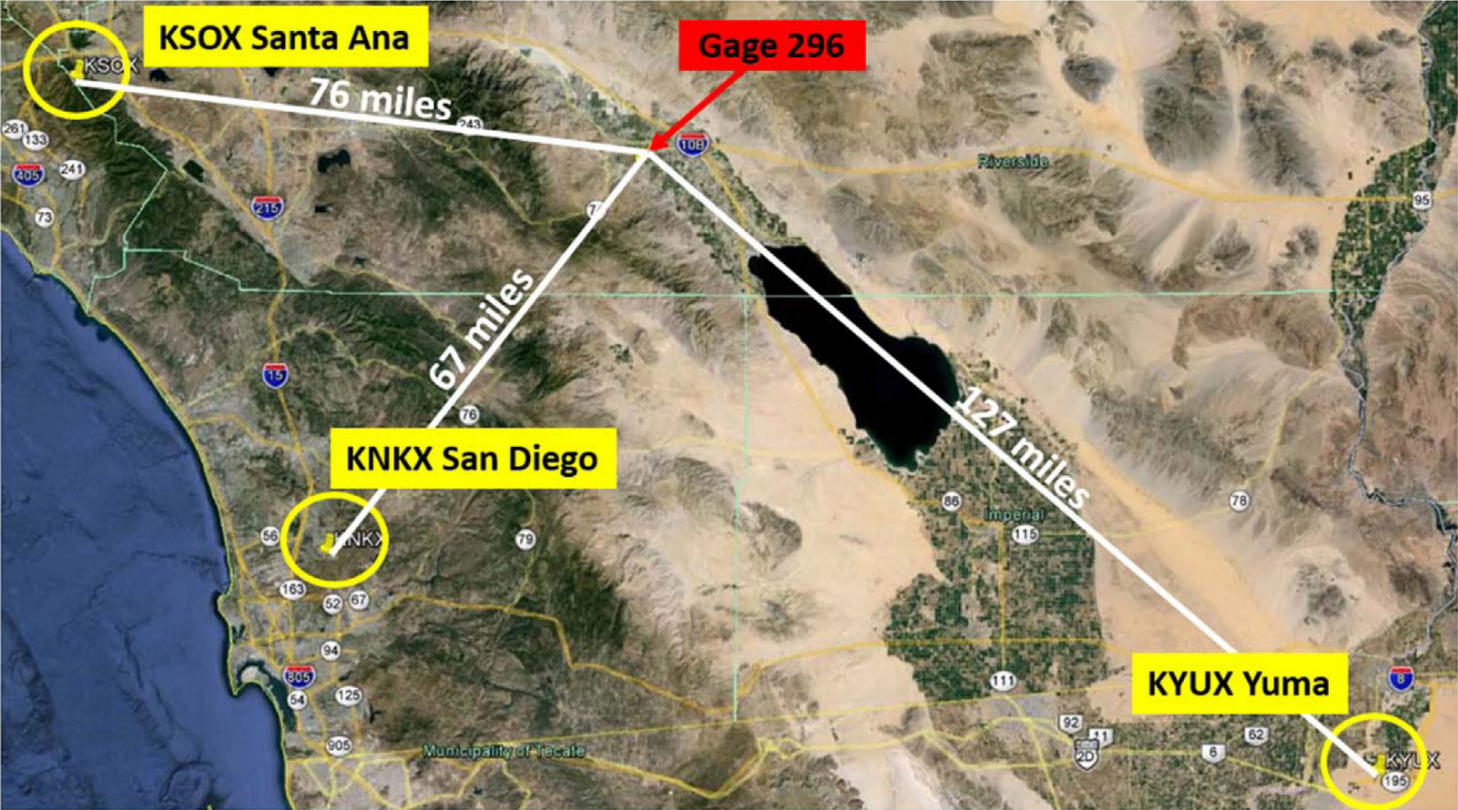
Radar station distances from subject rain gage.

Weather radar stations operate on a regional scale by emitting short bursts of radio waves and “listening” for the echo created by rain drops in the atmosphere. The radar antenna is usually set to scan 360°. Three radar stations [4] surrounding Gage 296 are shown in Fig. 2: KNKX San Diego (67 miles to the SW), KSOX Santa Ana Mountain (76 miles W) and KYUX Yuma (127 miles to the SE). The pictorial representation of the radar data is commonly used by news and weather stations and is frequently relied upon by the general public to visualize both the intensity and direction of a storm event. The radar data are recorded on a pixel basis. For each of the three radar stations, the radar data for the pixel which contained Gage 296 was examined.

## Comparison of Doppler radar estimates of rainfall versus measured rain gage rainfalls

### Storm Event #1 (August 25, 2013)

For the storm event of August 25, 2013, the rainfall recorded at Gage 296 and the data published for the three radar stations are summarized below.

**Fig. 3:**
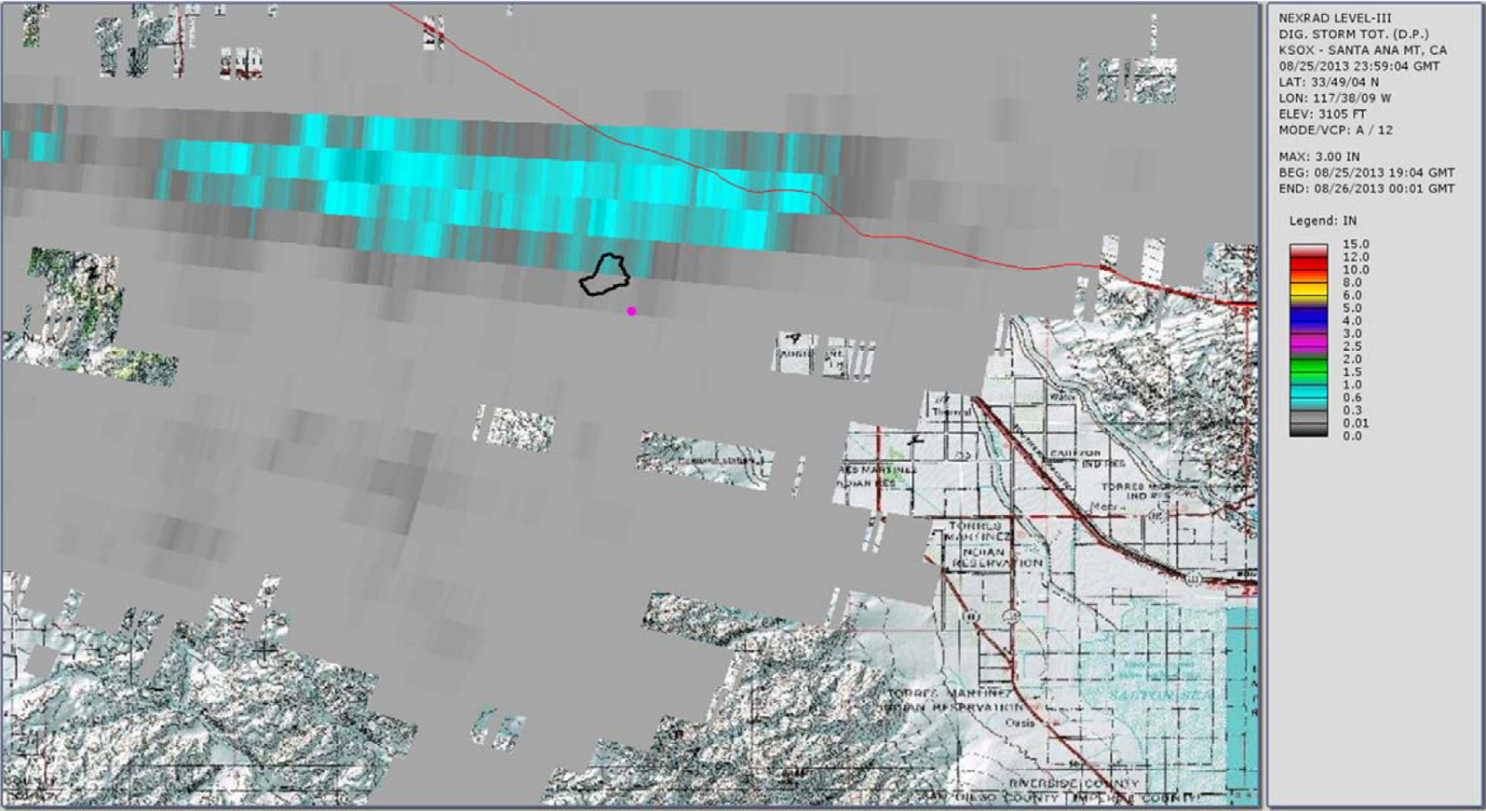
Radar station KSOX Santa Ana published rainfall estimate (storm total for storm event 8/25/13 at rain Gage 296 = 0.10 in.).

**Fig. 4:**
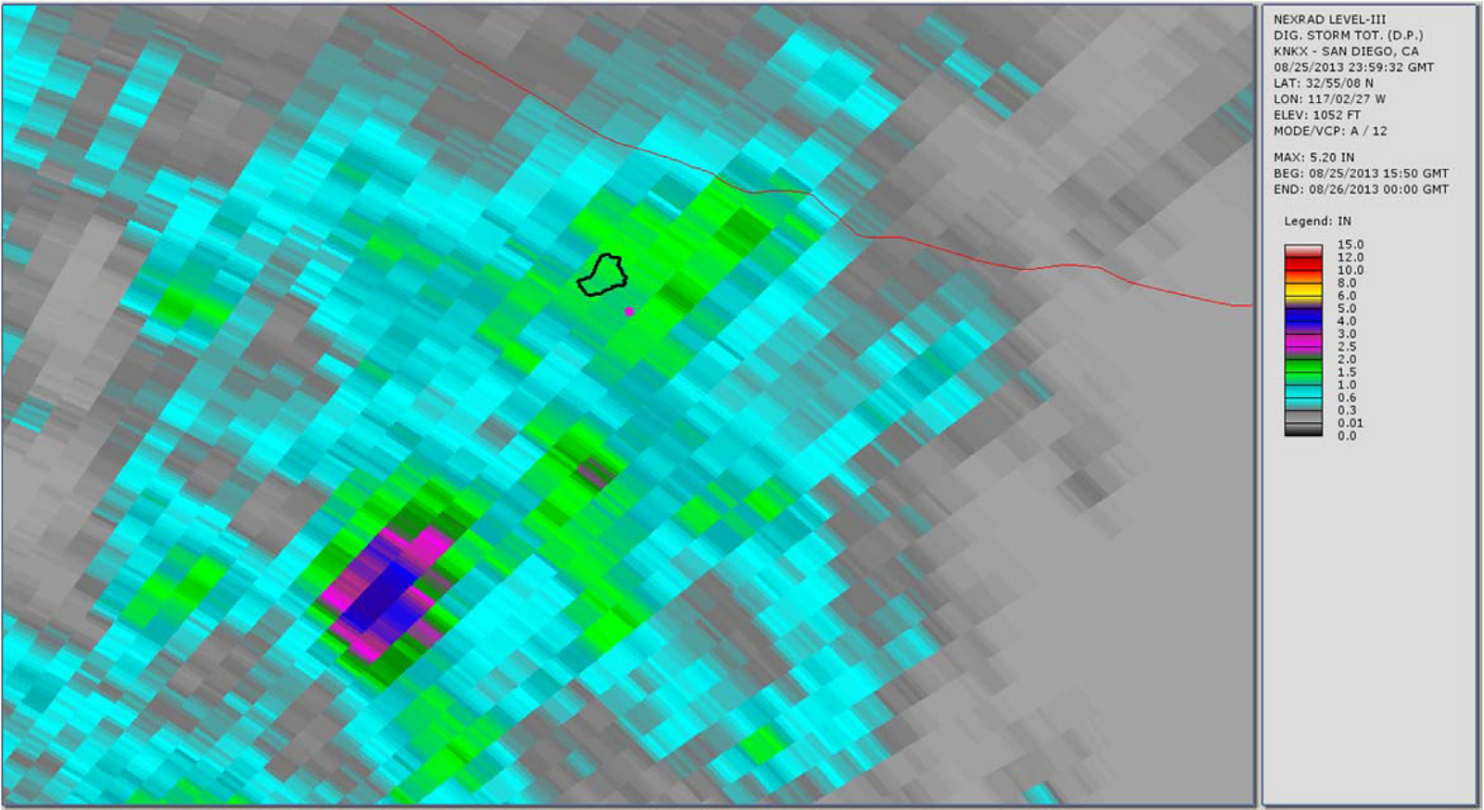
Radar station KNKX San Diego published rainfall estimate (storm total for storm event 8/25/13 at rain Gage 296 = 1.17 in.).

**Fig. 5:**
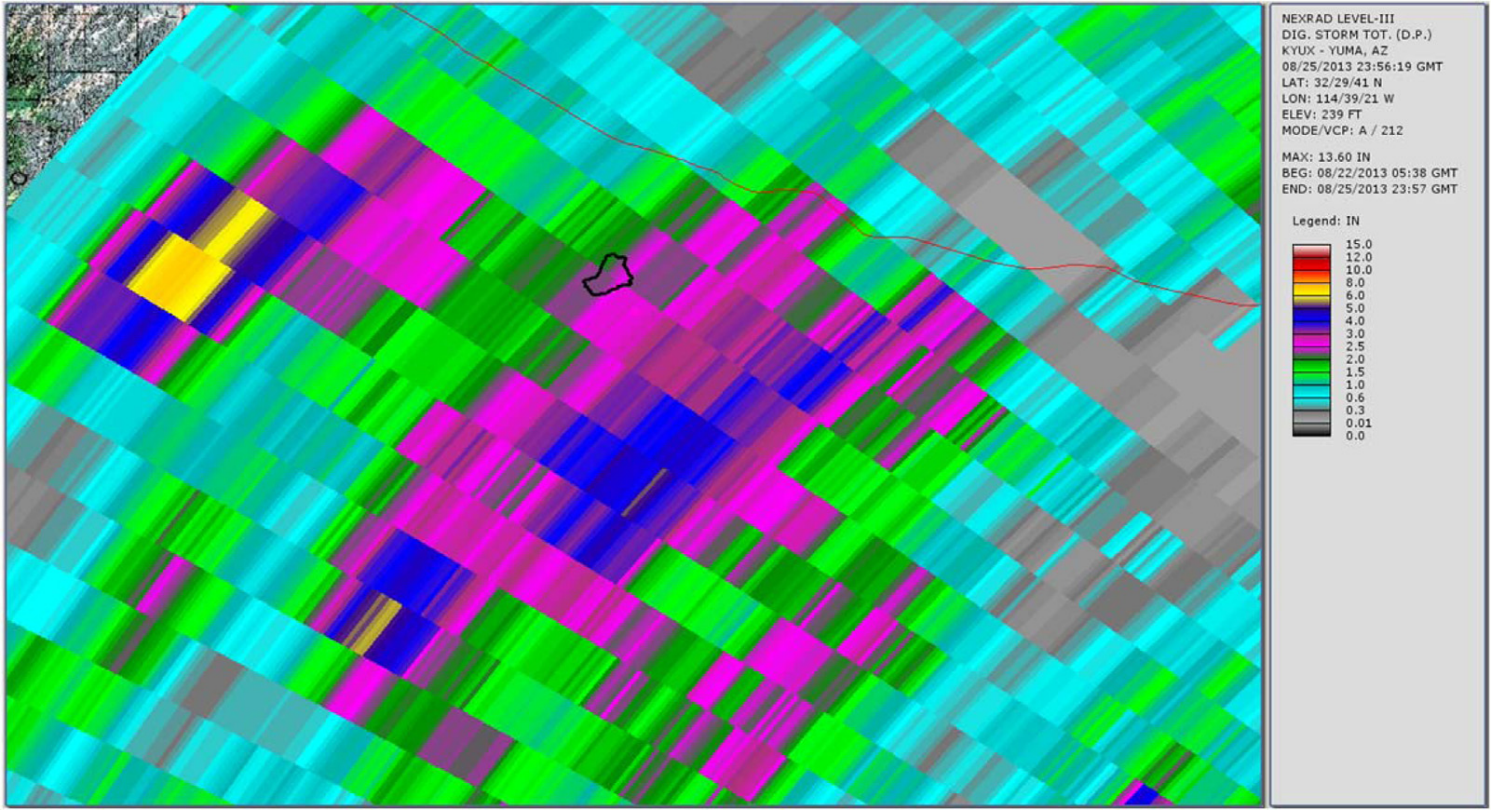
Radar station KYUX Yuma published rainfall estimate (storm total for storm event 8/25/14 at rain Gage 296 = 2.34 in.).

Fig. 3, Fig. 4, Fig. 5 show the common radar presentations of the daily rainfall for each of the three radar stations. Looking at the radar data in the table above and the pictorial presentations of that data in the figures below, a significant discrepancy is readily seen at the Gage 296 location. If one were to strictly rely upon the Doppler radar estimates of rainfall from KSOX Santa Ana, the storm of August 25, 2013 would appear to have been a minor event with 0.10 in. of rainfall falling in the peak hour. However, from the Doppler radar station KYUX Yuma published results, 2.09 in. of rainfall during the peak hour is indicated. In order to determine which radar station outcome gives a more accurate description of the storm event, a comparison is made of published rainfall estimates from each radar station to the actual rainfall data collected at a rain gage (Gage 296). This concept of “ground-truthing” radar data, by comparing it to actual rainfall data recorded at a gage, is well described in the literature. For this storm (see Table 1), we see that the KYUX radar station rainfall estimates correlate well to the rainfall data collected by Gage 296 for both the Storm Total for the day (midnight to midnight), as well as the Peak Hour measurements. (It is noted that the storm is essentially a 1 h duration event.)

### Storm Event #2 (September 8, 2014)

For the storm event of September 8, 2014, the rainfall recorded at rain Gage 296 and the rainfall estimates published for the three radar stations are summarized in Table 2 below.

**Fig. 6:**
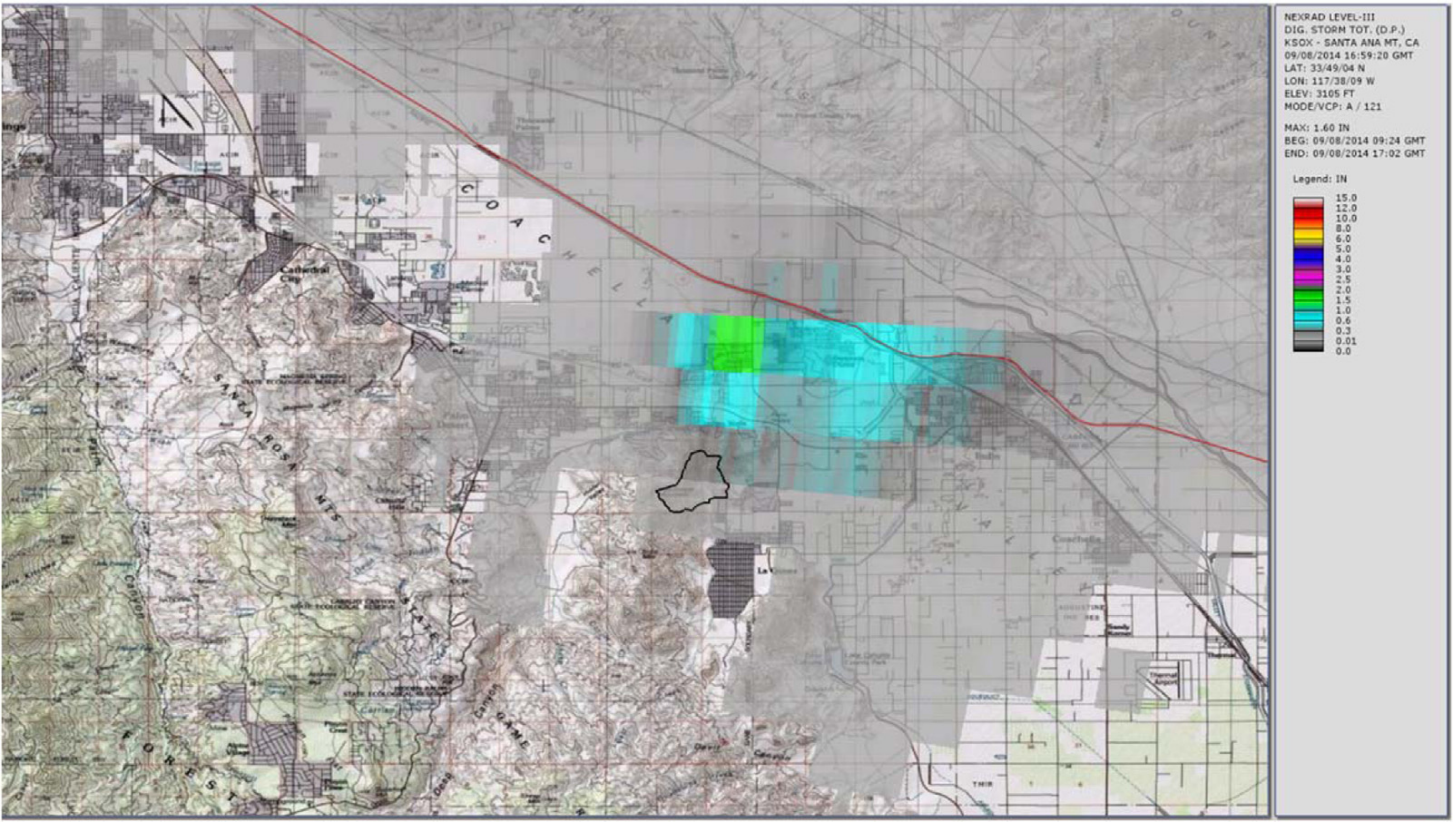
Radar station KSOX Santa Ana published rainfall estimate (storm total for storm event 9/8/14 at rain Gage 296 = 0.04 in.).

**Fig. 7:**
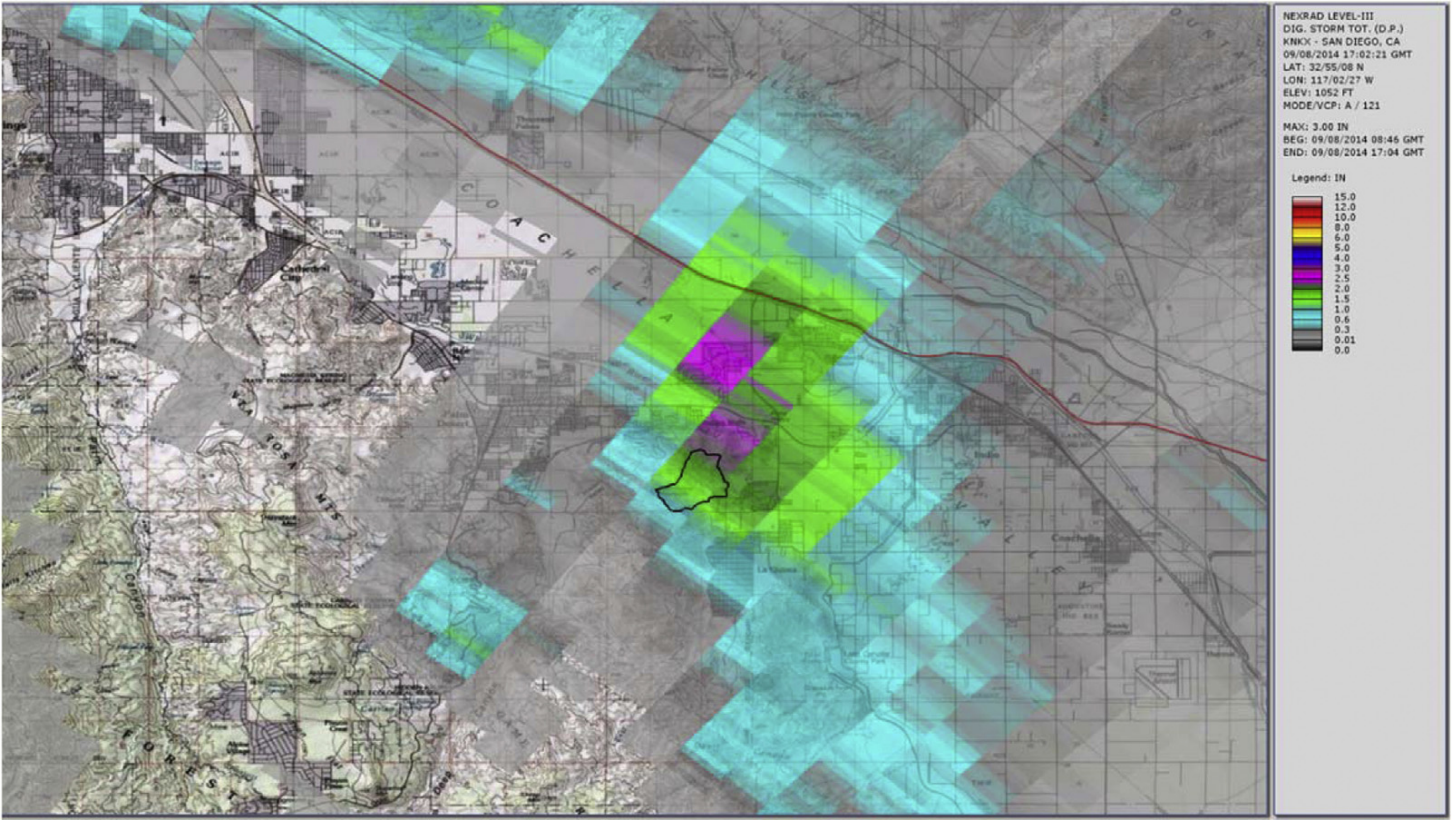
Radar station KNKX San Diego published rainfall estimate (storm total for storm event 9/8/14 at rain Gage 296 = 1.28 in.).

**Fig. 8:**
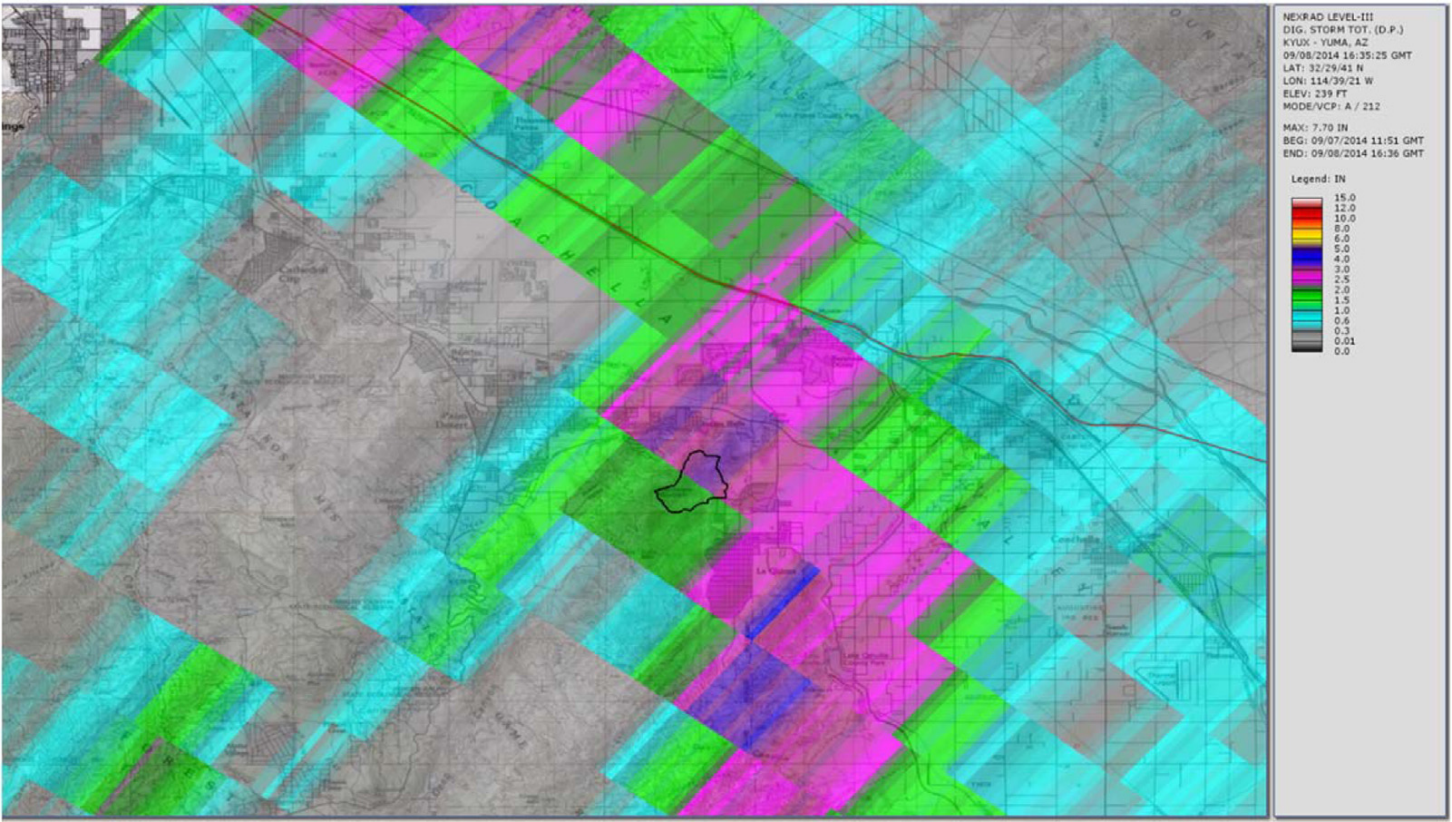
Radar station KYUX Yuma published rainfall estimate (storm total for storm event 9/8/14 at rain Gage 296 = 2.19 in.).

Fig. 6, Fig. 7, Fig. 8 show the usual radar graphical presentations of the daily rainfall for each of the three radar stations. Again, it is noted that the estimates of the amount of rainfall which fell at the Gage 296 location varies significantly, depending on which radar station is considered. Note that based on the Storm Total for the day (midnight to midnight), as well as the Peak Hour measurements, rain Gage 296 data seem to correlate well with radar station KYUX Yuma; however, rain Gage 296 overall records higher rainfall quantities than the KYUX Yuma radar station estimates indicate.

## Topographic interference profiles

**Fig. 9:**
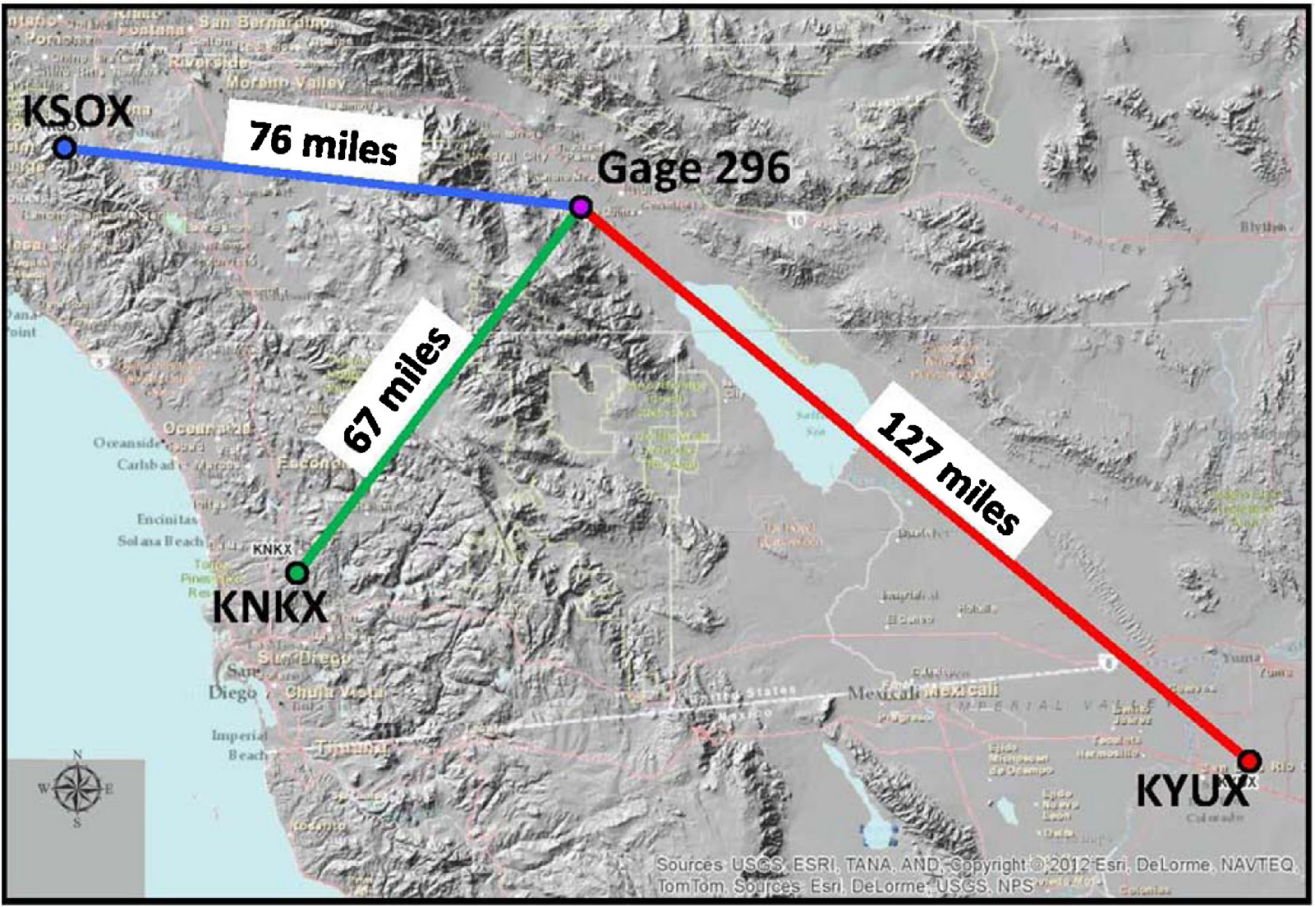
Radar station topographic locations.

**Fig. 10:**
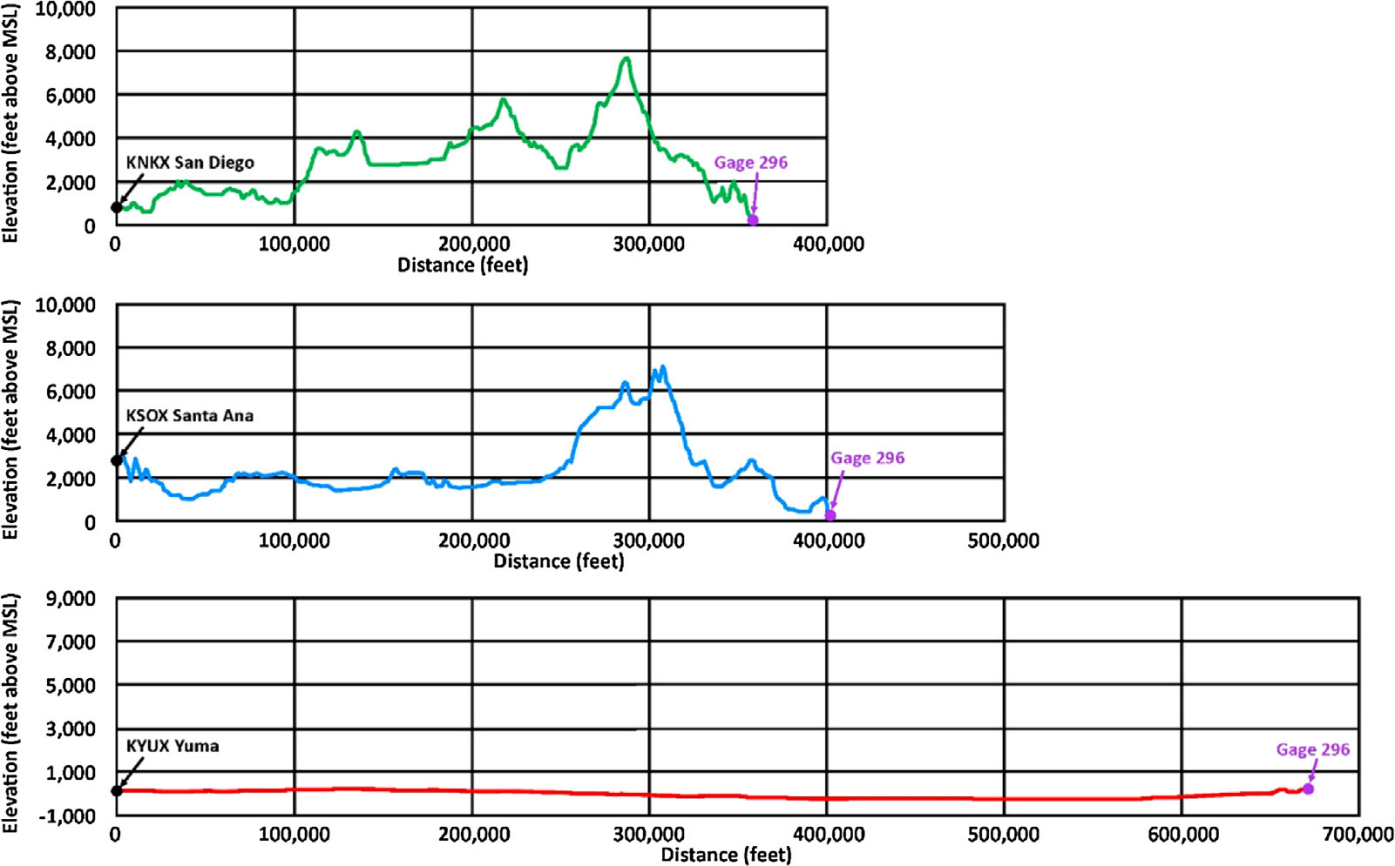
Radar station topographic profiles.

To understand the significant variation between the radar stations published estimates of rainfall, an examination is made of the topography located between the radar stations and the rain gage location. Fig. 9 shows the radar station locations with respect to the rain Gage 296. Fig. 10 depicts the topographic cross sections between each of the radar stations and the rain gage.

Note that for both the KNKX Santa Ana and KSOX San Diego radar stations, significant topographic interference exists between the radar stations and the rain gage location, whereas the elevation differential between radar station KYUX Yuma and rain Gage 296 is not significant. Recall that the radar station operates by emitting a series of short radio wave bursts and recording their echo off of rain drops in the atmosphere. Some of these radio waves are “interrupted” by the intervening mountains and therefore never reach the rain gage location to interact with the rainfall at that site. The published rainfall estimates that are recorded for the rain gage location by these two radar stations is limited to the rain drops in the upper atmosphere at an elevation higher than the intervening topography and may therefore not be an accurate representation of all of the rainfall occurring at rain Gage 296. In comparison, the radar station KYUX Yuma has no such topographic interference and can give a more complete representation of rainfall over the rain gage location.

## Ground truthing

Having concluded that the KYUX Yuma radar station data are more appropriate for comparing with the Gage 296 data, a correlation between these two sets of data, for two different severe intensity storm events, is made. The correlation seen between rain Gage 296 and radar estimates published for KYUX Yuma for the 8/25/13 storm event suggests that such a correlation exists for any rainfall event. However, this hypothesis of good correlation for all storm events is not established by the data from storm event 9/8/14. Recall that the radar antenna receives “echoes” from the interference of rain drops in the atmosphere. However, the radar antenna is limited in its broadcast “band”. Therefore, it is necessary to “ground truth” the radar station results by comparing the radar data to actual recorded rainfall at a rain gage in order to find the appropriate adjustment factor for each particular storm event. Using the peak hour data for the storm event of 8/25/13, this adjustment factor was approximately 1. For the peak hour data in the storm event of 9/8/14, the adjustment factor is approximately 1.42.

## Conclusions

In this paper, published Doppler radar results are compared with actual rain gage data for two severe short duration storm events that occurred within close proximity of each other and one year apart in time. Three Doppler radar sites are available in published rainfall estimations that can be compared directly with the rain gage data, and can be compared with respect to each other. The two storm events are both of high intensity short duration rainfalls occurring in a desert area of Southern California, USA. The occurrence of these two short duration storm events affords the opportunity to examine the accuracy in using published radar estimates of rainfall, and also examines the correlation of such radar estimates of rainfall to actual rainfall data for different storm events. It is concluded that consideration of possible topographic interference as well as ground-truthing of Doppler radar estimates of rainfall may be necessary for most storm event analysis based upon use of Doppler radar data.

## Figures and Tables

**Table 1:** Rainfall and Doppler radar rainfall estimates for the storm event of August 25, 2013.

	Rain Gage 296	KSOX Santa Ana	KNKX San Diego	KYUX Yuma
Storm total (inch)	2.32	0.10	1.17	2.34
Peak hour (inch)	2.08	0.10	1.06	2.09

**Table 2:** Rainfall and Doppler radar rainfall estimates for the storm event of September 8, 2014.

	Rain Gage 296	KSOX Santa Ana	KNKX San Diego	KYUX Yuma
Storm total (inch)	3.08	0.04	1.28	2.19
Peak hour (inch)	2.84	0.04	1.26	2.09
